# Effects of Apatite Cement Containing Atelocollagen on Attachment to and Proliferation and Differentiation of MC3T3-E1 Osteoblastic Cells

**DOI:** 10.3390/ma9040283

**Published:** 2016-04-13

**Authors:** Masaaki Takechi, Yoshiaki Ninomiya, Kouji Ohta, Misato Tada, Kazuki Sasaki, Mohammad Zeshaan Rahman, Akira Ohta, Kanji Tsuru, Kunio Ishikawa

**Affiliations:** 1 Department of Oral and Maxillofacial Surgery, Division of Cervico-Gnathostomatology, Programs for Applied Biomedicine, Graduate School of Biomedical Sciences, Hiroshima University, Hiroshima 734-8553, Japan; takechi@hiroshima-u.ac.jp (M.T.); yn@hiroshima-u.ac.jp (Y.N.); misatot@hiroshima-u.ac.jp (M.T.); sasaki@hiroshima-u.ac.jp (K.S.); md.zeshaan.rahman@gmail.com (M.Z.R.); akira.ota.114@gmail.com (A.O.); 2 Department of Biomaterials, Faculty of Dental Science, Kyushu University, Fukuoka 812-8582, Japan; tsuru@dent.kyushu-u.ac.jp (K.T.); ishikawa@dent.kyushu-u.ac.jp (K.I.)

**Keywords:** apatite cement, atelocollagen, osteoconductivity, MC3T3-E1 cell proliferation

## Abstract

To improve the osteoconductivity of apatite cement (AC) for reconstruction of bone defects after oral maxillofacial surgery, we previously fabricated AC containing atelocollagen (AC(ate)). In the present study, we examined the initial attachment, proliferation and differentiation of mouse osteoblastic cells (MC3T3-E1 cells) on the surface of conventional AC (c-AC), AC(ate) and a plastic cell dish. The number of osteoblastic cells showing initial attachment to AC(ate) was greater than those attached to c-AC and similar to the number attached to the plastic cell wells. We also found that osteoblastic cells were well spread and increased their number on AC(ate) in comparison with c-AC and the wells without specimens, while the amount of procollagen type I carboxy-terminal peptide (PIPC) produced in osteoblastic cells after three days on AC(ate) was greater as compared to the others. There was no significant difference in regard to alkaline phosphatase (ALP) activity and osteocalcin production by osteoblastic cells among the three surface types after three and six days. However, after 12 days, ALP activity and the produced osteocalcin were greater with AC(ate). In conclusion, AC(ate) may be a useful material with high osteoconductivity for reconstruction of bone defects after oral maxillofacial surgery.

## 1. Introduction

Hydroxyapatite (HAP: Ca_10_(PO_4_)_6_(OH)_2_) is widely used as a substitute material for the reconstruction of bone in oral maxillofacial surgery procedures [1,2]. However, a disadvantage of sintered HAP is difficulty with complete fitting of the shape to the bone defect [3]. Apatite cement (AC) was developed to overcome this disadvantage, as it sets to form HAP after mixing of the powder phase and liquid phase [4,5,6]. Thereafter, various types of AC were fabricated in an attempt to improve the properties in previous studies. Although conventional AC (c-AC) has some disadvantages, such as a long setting time of 30–60 min, fast-setting AC (fs-AC) can set within 5 min [7,8]. Anti-washout AC (aw-AC) is stable and sets even when the paste is immersed in serum immediately after mixing [9,10]. Our previous report demonstrated that these three types of ACs have essentially the same levels of biocompatibility and osteoconductivity for proliferation and differentiation of osteoblasts *in vivo* [3]. Based on those findings, we considered that the ability of these ACs as the substitutes for reconstruction after oral maxillofacial surgery require improvement to provide for higher amounts of osteoconductivity.

Collagen is a primary component of bone and contributes mechanical properties that maintain the strength and structural integrity [11]. Furthermore, collagen has biological properties and demonstrates effects on cell differentiation, adhesion, growth and migration [12]. Atelocollagen is a type of soluble collagen produced from tropocollagen, the collagen molecule that makes up collagen fibrils, via the elimination of telopeptide moieties, which are considered to account for most of collagen’s antigenicity [13].

In our previous study, we fabricated AC containing atelocollagen (AC(ate)) and examined soft tissue responses to AC and AC(ate) *in vivo*. Those results showed a good soft tissue response in inflammatory conditions by AC(ate) as compared to c-AC [14]. However, it remained unclear whether AC(ate) has higher osteoconductivity than c-AC. In this study, we examined initial cell attachment, proliferation and differentiation of mouse osteoblastic cells (MC3T3-E1 cells) cultured on the surface of c-AC, AC(ate), as well as plastic cell culture wells used as a control.

## 2. Results

**Figure 1:**
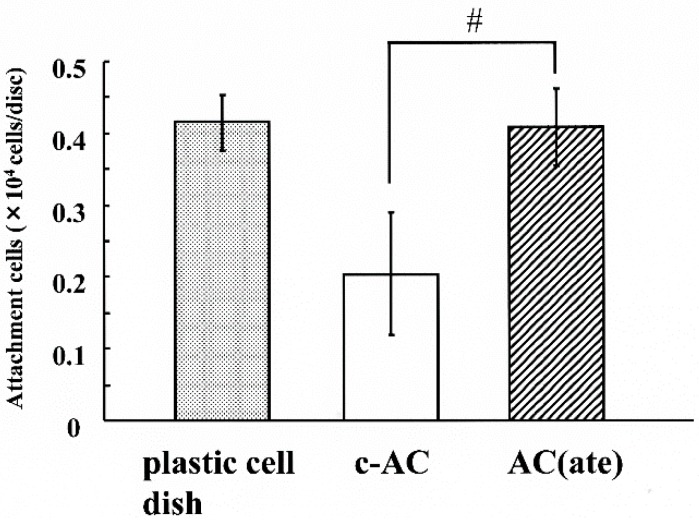
Initial attachment of cells to the surface of conventional apatite cement (c-AC), AC containing atelocollagen (AC(ate)) and plastic wells. The numbers of MC3T3-E1 cells with all of the specimens were determined after 5 h of culturing. Results are shown as the mean ± standard deviation (*n* = 5). # Significant difference as compared to c-AC (Student’s *t*-test: *p* < 0.05).

**Figure 2:**
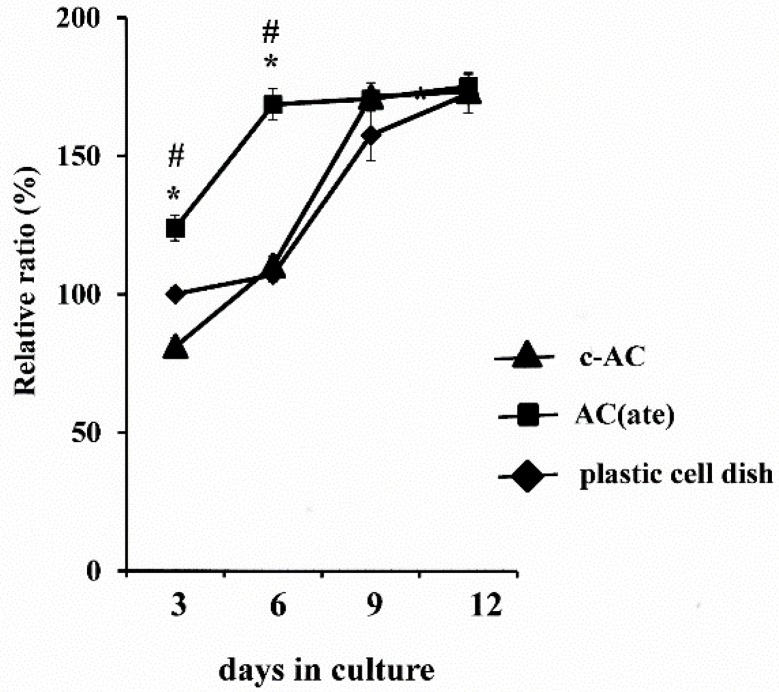
Proliferation of MC3T3-E1 cells on the surface of each type of specimen as evaluated by a methyl thiazolyl tetrazolium (MTT) assay. The results are shown as the mean ± standard deviation (*n* = 5). * and # indicate a significant difference as compared to plastic cell well and c-AC, respectively (Student’s *t*-test: *p* < 0.05).

**Figure 3:**
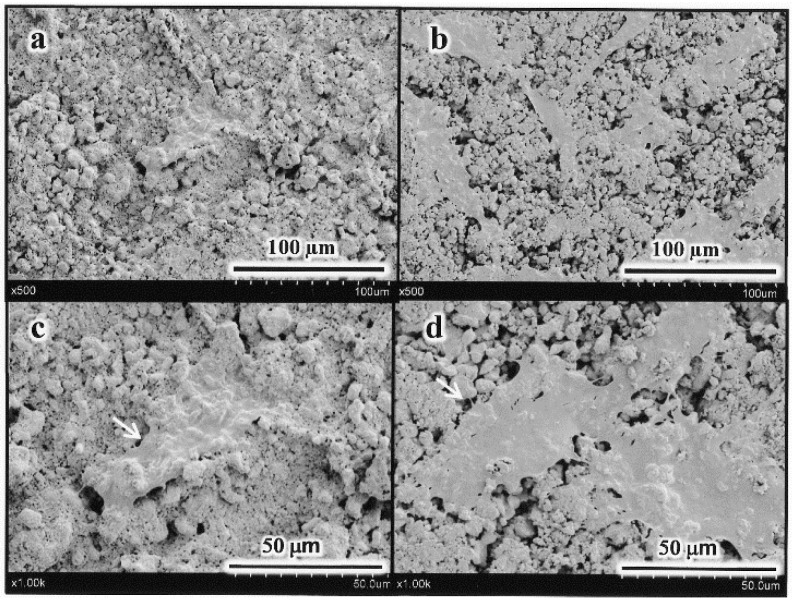
Typical scanning electron microscope (SEM) images of the surfaces for (**a**,**c**) c-AC and (**b**,**d**) AC(ate) after cell culturing for three days. Original magnification lower (**a**,**b**) and higher (**c**,**d**).

**Figure 4:**
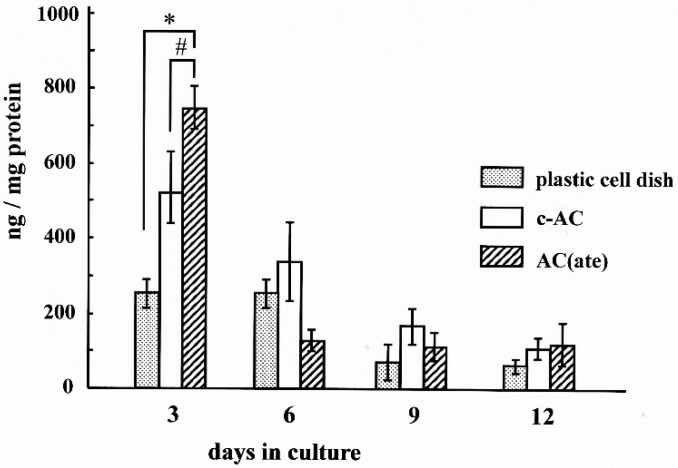
Time course of the amount of procollagen type I carboxy-terminal peptide (PICP) produced by MC3T3-E1 cells in cultures with each type of specimen. Results are shown as the mean ± standard deviation (*n* = 5). * and # indicate a significant difference as compared to the plastic cell well and c-AC, respectively (Student’s *t*-test: *p* < 0.05).

**Figure 5:**
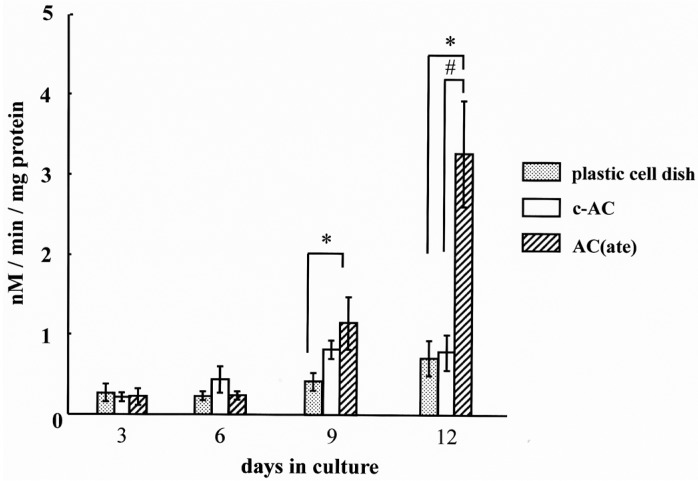
Time course of alkaline phosphatase (ALP) activity by MC3T3-E1 cells in cultures with each type of specimen. Results are shown as the mean ± standard deviation (*n* = 5). * and # indicate a significant difference as compared to the plastic cell well and c-AC, respectively (Student’s *t*-test: *p* < 0.05).

**Figure 6:**
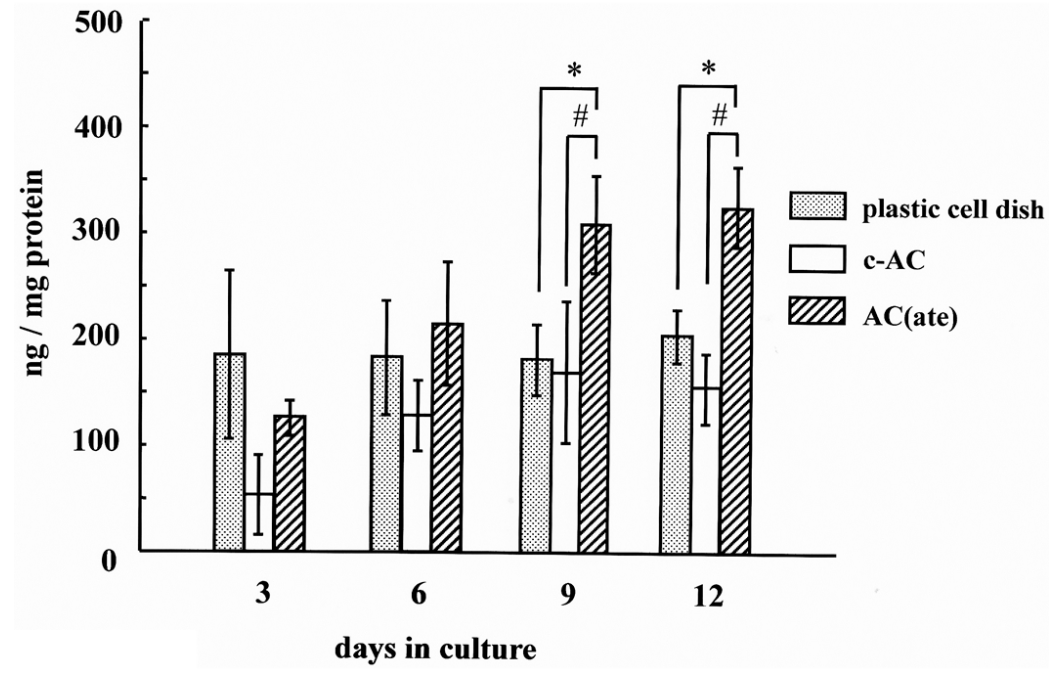
Time course of the amount of osteocalcin produced by MC3T3-E1 cells in cultures with each type of specimen. Results are shown as the mean ± standard deviation (*n* = 5). * and # indicate a significant difference as compared to the plastic cell well and c-AC, respectively (Student’s *t*-test: *p* < 0.05).

We found that the number of MC3T3-E1 cells initially attached to AC(ate) is greater than that of those to c-AC and was similar to the number attached to the plastic cell wells in Figure 1. Figure 2 shows that the number of MC3T3-E1 cells on the surface of each sample increased with incubation time, with those attached to AC(ate) rapidly increasing from Days 3–6, as compared to c-AC and the plastic wells. The adhered cells on the c-AC and AC(ate) after three days showed the typical osteoblast morphology, and these cells on AC(ate) were well spread as compared to those on c-AC, as shown in Figure 3. The cultured cells on AC(ate) were almost confluent in the cement bulk after six days (data not shown). The production of procollagen type I carboxy-terminal peptide (PIPC) and osteocalcin and the alkaline phosphatase (ALP) activities of MC3T3-E1 cells were determined as markers of differentiated osteoblasts. Figure 4 shows that the level of PIPC on AC(ate) was greater as compared to the plastic wells and c-AC on Day 3, after which PIPC production in all specimens decreased. However, on Day 12, the PIPC level with AC(ate) remained dramatically higher. There was no significant difference for ALP activity in the MC3T3-E1 cells among the three specimen types on Days 3 and 6, though a larger increase was found with AC(ate) as compared to the others on Day 12 in Figure 5. Similarly, as shown Figure 6, no significant difference in osteocalcin production was noted among the specimens on Days 3 and 6, while AC(ate) showed a greater increase as compared to the others from Days 9–12.

## 3. Discussion

Atelocollagen is a type of collagen obtained from antigenic telopeptides and considered to be preferable for biomaterial use as compared to other types of collagen, because of its low level of antigenicity [11]. Collagen has been reported to accelerate the regeneration and osteoconduction of cementum when used as a coating material with calcium phosphate ceramics and tricalcium phosphate [15]. Miyamoto *et al.* fabricated AC containing atelocollagen and examined the basic properties of AC with atelocollagen content from 0.5% to 5%. They found that 2% content or over of atelocollagen improved the handling properties, was more adhesive and is desirable for its use in surgical procedures. However, the addition of atelocollagen to AC has disadvantages, such as a decrease of diametral tensile strength (DTS) and an increase of setting time [16]. From those results, the optimal additional concentration of atelocollagen in AC for surgical applications was decided to be 2%, and our group investigated the response of soft tissue and bone to AC containing 2% atelocollagen (AC(ate)) *in vivo* and found that the inflammatory reaction around AC(ate) in rat abdominal skin was lower than that around c-AC, while new bone formation around AC(ate) by rat tibia bone was greater than that observed around c-AC [14]. In the present *in vitro* study, we demonstrated that initial attachment, proliferation and differentiation of osteoblastic cells on AC(ate) were greater as compared to c-AC.

Initial cell attachments, as well as proliferations on an AC surface are important factors for the osteoconduction in bone defects. In our previous studies, we noted that the number of cells initially attached to c-AC, fs-AC and aw-AC was lower as compared to plastic cell wells, whereas the cell proliferation among those specimens was similar [3]. In the present study, the number of cells showing initial attachment to AC(ate) was greater as compared to AC and similar to the numbers attached to the plastic wells. Thereafter, the number of cells on AC(ate) rapidly increased as compared to c-AC and the wells. Collagen specifically binds cell-surface cellular adhesion molecules, such as integrins, which enhance the cell adhesion and proliferation of osteoblasts via various signal transduction factors [17]. Furthermore, collagen may also function in a matrix in which particles of calcium phosphate ceramic, such as hydroxyapatite, are anchored [14]. We concluded that AC(ate) may promote osteoblastic adhesion and proliferation from host bone in bone defects, along with providing the basic properties of atelocollagen.

Procollagen type I carboxy-terminal peptide (PICP), ALP and osteocalcin are known as markers of osteoblast cell differentiation. PICP is released from osteoblasts during the early differentiation stage when type I collagen is synthesized into bone [18], while ALP and osteocalcin are mid- and late-stage markers, respectively, of cell differentiation [19]. Previously, our group found that the levels of these differentiation markers in cultures with c-AC, fs-AC and aw-AC were similar, while they were higher than cultures with a plastic dish and sintered-HAP at each stage, indicating that these AC formulations have a greater ability to enhance the differentiation of osteoblasts [3]. Type 1 collagen promotes various processes during osteoblast differentiation, such as ALP activity, expression of non-collagenous extracellular matrix proteins and deposition of minerals into the matrix [12]. In the present study, we found that the expression of cell differentiation markers in cultures with AC(ate) were significantly higher as compared to AC and the plastic wells at each stage, indicating an interaction among HAP, bone mineral components, the bone extracellular matrix and collagen to accelerate osteoblastic cell differentiation. On the other hand, collagen and HAP enhance the morphological differentiation of osteoclasts [20], while the extracellular matrix containing collagen fibers formed by osteoblasts was reported to promote osteoclastic resorption of calcium phosphate ceramics *in vitro* [21]. Therefore, AC(ate) may augment bone regeneration by promoting differentiation of host osteoblasts and osteoclasts during the remodeling process, indicating its usefulness as a material with high osteoconductivity for reconstruction of bone defects after oral maxillofacial surgery.

## 4. Materials and Methods

### 4.1. Preparation of AC Containing Atelocollagen

The powder phase of AC(ate), an equimolar mixture of tetracalcium phosphate (TTCP: Ca_4_(PO_4_)_2_O) and dicalcium phosphate anhydrous (DCPA: CaHPO_4_), was prepared as described previously. Neutral sodium hydrogen phosphate (pH 7.4) was made by mixing 0.2 mol/L Na_2_HPO_4_ with 0.2 mol/L NaHPO_4_, with the resulting solution showing pH 7.4 at 37 °C. A 2% atelocollagen solution (atelocollagen, from calf corium, Koken, Tokyo, Japan) containing those Na_2_HPO_4_ solutions was used as the liquid phase of AC(ate). As for c-AC, we used distilled water as the liquid phase. The powder and liquid phases were mixed with a spatula at a powder to liquid ratio (P/L ratio) of 3.5. The obtained paste was packed into a plastic mold (diameter of 10 mm) at a pressure of approximately 1.0 MPa under constant vibration (Automatic Labo-Mixer NS-8, Iuchi, Osaka, Japan) and allowed to harden in an incubator at 37 °C with 100% relative humidity for 24 h. The discs were then polished to a thickness of 200 μm.

### 4.2. Cells and Incubation Condition

MC3T3-E1 cells were used as previously described [3] and cultured in alpha-modified minimum essential medium (Dainihonseiyaku, Osaka, Japan) supplemented with 50 μg/mL ascorbic acid, 2 mmol/L β-glycerophosphate (Sigma Chemical Co., St. Louis, MO, USA) and 10% fetal bovine serum (Whittaker Bioproducts, Inc., Walkerville, MO, USA). Each disc was placed in a plastic well (48-well cell-cultured cluster, Corning, NY, USA); then, 500 μL of culture medium containing a cell suspension at concentration of 1 × 10^3^ cells per 10 mm disc was gently poured onto each disc, as well as into empty plastic wells as the control. After 5 h, 2 mL of medium were added to each well, and the samples were incubated in an atmosphere containing 5% CO_2_ at 37 °C.

### 4.3. Initial Cell Attachment and Proliferation of MC3T3-E1 Cells with c-AC and AC(ate)

The number of MC3T3-E1 cells attached to each disc or empty well was determined as follows. Following the initial 5-h incubation, the specimens were washed with 0.1 mol/L phosphate buffer saline (PBS) to eliminate non-adherent cells, then adherent osteoblasts were harvested using PBS containing 0.1% collagenase and trypsin and counted using a hemocytometer.

Cell proliferation was then determined after 3, 6, 9 and 12 days of incubation using a methyl thiazolyl tetrazolium (MTT) assay. MTT reagent (3-[4,5-dimetylthiazole-2-yl]-2,5-diphenyltetrazolium bromide), which is enzymatically converted by living cells into a blue/purple formazan product, was added to each sample and incubated at 37 °C for 4 h. The blue formazan product was solubilized with dimethylsulfoxide, and the liquid portion of each sample was removed for the assay, which was performed in a 96-well plate. Absorbance was read using a microplate reader (MTP-32 Microplate Reader, Corona elect, Ibaragi, Japan) at 570 nm.

The cell morphology adhered on the specimens was observed by a scanning electron microscope (SEM, S-3400N, Hitachi High-Technologies Co., Tokyo, Japan) at 5 kV of accelerating voltage after the fixation and dehydration followed by gold–palladium coating (magnetron sputtering machine; MSP-1S, Vacuum Device Co., Ibaraki, Japan).

### 4.4. Alkaline Phosphatase Activity in MC3T3-E1 Cells with c-AC and AC(ate)

The ALP activity of the osteoblasts was determined using an enzyme-linked immunosorbent assay (ELISA) with p-nitrophenyl phosphate as the substrate (ALP substrate system, Kirkegaard & Perry Lab., Gaithersburg, MD, USA). The absorbance of *p*-nitrophenol at 405 nm was determined with a plate reader (MTP-32 Microplate Reader, Corona elect, Ibaragi, Japan). Enzymatic activity is expressed as nmol of p-nitrophenol/minute per mg of protein. Protein content was determined by the method of Bradford using bovine r-globulin as the standard [22].

### 4.5. Production of Procollagen Type I Carboxy-Terminal Peptide and Osteocalcin in MC3T3-E1 Cells with c-AC and AC(ate)

PICP and osteocalcin were analyzed according to the method of Yuasa *et al.* [3]. Each specimen was decalcified with 0.6 N HCl for 30 min, then the resulting solutions were harvested and stored at −20 °C until the assay. The amounts of PICP and osteocalcin were measured using a Procollagen Type I C peptide EIA kit and Osteocalcin EIA kit, respectively (Takara, Shiga, Japan). Each experiment for measurements of ALP activity, PICP protein levels and osteocalcin level was performed in triplicate and repeated 3 times.

### 4.6. Statistical Analysis

Data were analyzed using Student’s *t*-test or one-way analysis of variance (ANOVA), and the results are presented as the mean ± standard deviation.

## 5. Conclusions

The addition of atelocollagen to AC has been reported to provide some advantages, such as improvement in handling properties and adhesive properties when used for surgical applications, and soft tissue showed a good inflammatory response. In the present study, AC(ate) increased initial attachment, proliferation and differentiation of osteoblasts in comparison with c-AC. We consider AC(ate) to be a useful material with high osteoconductivity for reconstruction of bone defects after oral maxillofacial surgery.
